# Supplementary material for: An evolutionary approach to predict the orientation of CRISPR arrays
Source: PLoS Comput Biol. 2025 Nov 18;21(11):e1013706. doi: 10.1371/journal.pcbi.1013706 (PMC12638026; doi:10.1371/journal.pcbi.1013706)
Supplement: Attachment — Submitted filename: crisprevor_letter_to_reviewers.pdf [file pcbi.1013706.s003.pdf]

Title:
An evolutionary approach to predict the orientation of CRISPR arrays

Authors:
Axel Fehrenbach, Alexander Mitrofanov, Omer S. Alkhnbashi, Rolf Backofen and Franz Baumdicker

Dear reviewers,
thanks for your careful reading of the manuscript and the helpful comments and suggestions. We are confident that we could address all of your comments and hope to have clarified all open issues. We believe your comments and suggestions enabled us to significantly improve this manuscript. Below is a detailed list of answers to the points you raised. Note, that we provided a file that highlights the changes we made in the manuscript.

Reviewer #1
This study addresses the problem of determining the correct orientation of CRISPR arrays, which is essential for understanding their function and reconstructing their evolutionary history. Existing tools often rely on features such as leader sequences, PAMs, Cas genes, or repeat mutations, which can be inconsistent or absent. The authors present CRISPR-evOr, a new algorithm that infers orientation by reconstructing the evolutionary history of closely related CRISPR arrays in both directions. By comparing their likelihoods, they determine which orientation is more likely.
This work/algorithm is built upon their previous work: Fehrenbach, Axel, et al. "SpacerPlacer: ancestral reconstruction of CRISPR arrays reveals the evolutionary dynamics of spacer deletions." Nucleic Acids Research 52.18 (2024). It's implemented as a function of SpacerPlacer. Essentially, this work used SpacerPlacer's concept but applied to a unique problem.
The concept is interesting. The core novelty is calculating both forward and reverse scenarios and using a log-likelihood ratio to assess which direction is more plausible. One of the technical concepts is how to define a confidence threshold for making reliable orientation predictions.
Answer: We appreciate your feedback and thank you for your careful reading of the manuscript.

While I found the conceptual framework interesting, the manuscript is poorly written and structured. I found it very confusing to read and grasp the idea, and the figures need to be better designed to demonstrate the concepts. Also some of the key ideas need to be explained. Please see my specific comments.
Answer: We appreciate this comment and improved the figures, structure and general readability of the manuscript and addressed all of your comments as outlined below.

Major issues

1. Introduction, lines 133–134, right column, on the "low-confidence" areas, the statement "Thus, our tool increases the number of arrays where orientations can be reliably estimated" should be more informative with a quantitative performance comparison. This is a main problem with the maintext, it's more descriptive than quantitative.

A: *We adjusted the text to be more quantitative where possible. We added an additional line above the sentence you reference to give the actual percentage of arrays we are able to predict confidently that other tools can not (ll. 205 – 211):*

> We apply *CRISPR-evOr* on a large database of CRISPR arrays extracted from CRISPRCasdb [27] and find that *CRISPR-evOr* confidently predicts orientations for arrays where the aforementioned orientation methods have low confidence. Quantitatively, *CRISPR-evOr* can predict the orientation of 28.3% of all arrays where CRISPRDirection and CRISPRstrand have low confidence.

2. In Methods, the authors said "These arrays were grouped successively by repeat, genus, Cas type to yield a large comprehensive dataset." "Group" is a critical concept for the

evolution tree construction. But it is not sufficient explained, and too abstract to evaluate the algorithm built on this. Including an example could be helpful. Perhaps as part of Figure 2.

A: *We agree with you that the group clustering is crucial for evolutionary analysis. Both Figure 2 (former Figure 1) and Supplementary Fig. S2, S3 show examples of such groups and the respective captions state which species and Cas type are part of the groups. The most restrictive step of the group clustering are excluding low confidence arrays and clustering by spacer overlap. The remaining restrictions are done mainly to make the groups more homogeneous avoiding biases and random spacer overlap. The dataset was created for the* SpacerPlacer *paper and is clearly described in the original reference, with additional detail in Supplementary Note 1A. We would prefer to not simply reiterate the information in this manuscript. We adjusted the text to reference more clearly to respective sections in the* SpacerPlacer *paper. Incorporating your feedback we adjusted the description of the dataset as follows (ll. 232 – 255):*

> We rely on the CRISPRCasdb dataset collected by us in [13]. It is based on high-confidence arrays extracted from CRISPRCasdb [27] in July 2022. For each array the orientation prediction by CRISPRDirection was extracted from CRISPRCasdb and CRISPRstrand was applied to obtain the respective orientation predictions. These arrays were grouped successively by consensus repeat, spacer overlap, genus and Cas type to yield a large comprehensive dataset. For the grouping by spacer overlap, spacers were clustered as described in [13]. Then groups were removed if they did not share any spacer overlap between arrays or if their arrays were completely identical. Thus, each group contains only arrays of the same consensus repeat, genus, Cas type and overlapping spacer content. Examples of such groups are shown in Figure 2, Supplementary Fig. S2, S3. The dataset contains a total of 518 groups with 5934 arrays. A breakdown of the Cas types in the dataset is shown in Supplementary Fig. S1. Figure 4 shows the Cas type distribution for arrays with high confidence orientation predictions by *CRISPR-evOr*, CRISPRstrand and CRISPRDirection in this dataset.
>
> For more details about the preparation of the groups and the dataset in general, see Methods and Supplementary Note S1A in [13].

3. The left panels of Figure 1A, B could use a larger font size. Besides, I found this figure less essential to the main concepts and suggest removing it or move to supplementary material, and use the current figure 2 as figure 1, as it captures the concept of the algorithm. In figure 2, the author should label which step is done by which part of the algorithm.

A: *We agree with you and adjusted the order of Figure 1 and 2, i.e. Figure 1 is now Figure 2. We slightly adjusted Figure 1 (previously Figure 2) and labeled which steps are done by SpacerPlacer as you suggested. Moreover, we increased the font size of Figure 2 (previously Figure 1) by consolidating the events on the tree and shortening the strain names. Thank you for this comment. We believe the new versions of the Figures are a substantial improvement.*

4. The authors mentioned using SpacerPlacer in the Introduction but left it unexplained. It is also not mentioned that this comes from the same authors. Explaining the previous work is important, it will help the readers understand the novelty of this work and how it's tied to the rest of the field and the authors' research goal.

A: *We adjusted the introduction to give a short description of* SpacerPlacer *and to clearly state that this method was introduced by us (ll. 183 – 189):*

> For the reconstructions, we rely on our recently introduced tool *SpacerPlacer* [13]. *SpacerPlacer* reconstructs the ancestral history of spacer acquisitions and deletions of a group of arrays (with spacer overlap) along a phylogenetic tree by using

maximum likelihood methods and leveraging the spacer insertion order (inferred from the arrays).

5. The Result section is a mixture of methods+results+discussion. Please condense and move some of the content to other sections. "Orientation algorithm", "Description of orientation algorithm", "Likelihood comparison and measuring confidence" should be condensed into one subsection, i.e. the algorithm. Part of "Simulation-based benchmarking." And "Evaluation of acquisition-orientation on real data" should be moved to methods, important results can be kept.

A: *We consolidated the section as suggested and moved fitting parts to Methods or Discussion. The section "Simulation-based benchmarking" was moved to Methods.*

6. The work used several datasets, such as CRISPRCasdb and simulated, and benchmarked CRISPR-evOr against existing tools showing good performance in both simulated and real datasets. Please help the readers how do they look like and your data diversity. could use a figure.

A: *Both the CRISPRCasdb and PAM-orientation dataset have detailed description of their diversity in the respective papers in which they were introduced. All datasets represent snapshots of all available arrays available at the time from CRISPRCasdb/NCBI with large diversity in Cas types. In our manuscript the Cas type diversity for the CRISPRCasdb dataset is shown in Supplementary Fig. S1 and (indirectly) in Figure 4 for arrays with high confidence orientation predictions by the repeat-based tools and* CRISPR-evOr*. For the PAM-orientation dataset, the Cas type diversity is shown (indirectly) in Figure 3. Moreover, Supplementary Fig. S8 and S9 show confidence distributions of the discussed tools and S10 - S15 show Cas type diversity with respect to the pairwise comparison of predictions and confidence of all tools. The simulation datasets are described in Methods and are described in detail in the supplementary material. The simulation process is described in detail in the* SpacerPlacer *paper. We agree that this information was previously hard to identify in our manuscript. Thus we added additional references to the relevant Figures in section "Database and available orientation predictions" in Methods and where applicable in the manuscript.*

7. The threshold c is chosen empirically, based on simulation and manual inspection. While this approach is understandable for exploratory work, I believe a more formal justification is necessary, such as hypothesis testing, statistical modeling, or heuristic optimization. Please also discuss how this problem has been solved by the broader research community, not necessarily on this topic, but evolution, longitudinal data in general.

A: The choice of c is indeed a difficult problem since we do not expect that the likelihood ratio follows a canonical distribution. There are no canonical applicable tests or modeling methods to determine the distribution and such a threshold. Moreover, this threshold can not be identified by stress testing in simulations that follow the (ideal) model of polarized acquisitions with block deletions, since the method is almost 100% reliable in the simulation case (with $c = 0$*), even in more difficult settings (e.g. without a known tree, more realistic settings with very varied underlying parameters). However, in the case of the CRISPRCasdb dataset, we observed that for log-likelihood ratios above 5 (which indicates that the likelihoods differ by more than two orders of magnitude (factor of 148)) the differences between the reconstructions become substantial and the optimal orientation (assuming polarized insertion) becomes very clear. To reinforce this observation, we added an additional Supplementary Figure S4 that shows the difference in number of spacer deletions between the two reconstructions vs the modulus of the log-likelihood ratio. Clearly, the number of spacer deletions increases quickly with increasing ratio and decisions are thus based on an increasing number of (larger) deletion events. However, the threshold should be treated with caution and adapted when necessary. We note in Results (ll. 524 – 541):*

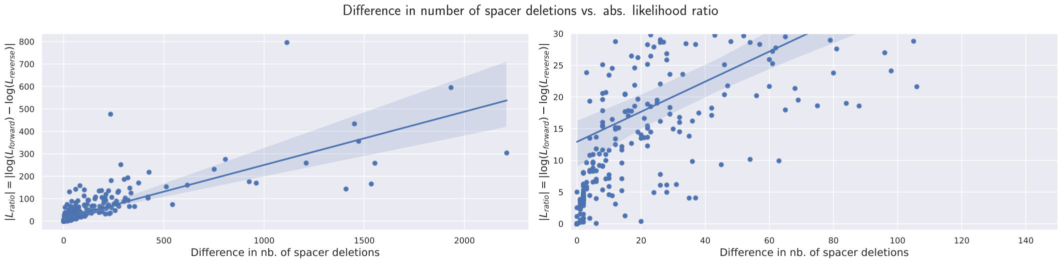

Supplementary Fig. S4: We show the fraction of confident predictions for each group and array-based tool versus the number of arrays in the respective group on the complete CRISPRCasdb dataset. Moreover, we show the means of the fraction of confident predictions for each group size and tool as the respective colored dashes. Note that we introduced some noise to the individual group percentages to make individual groups distinguishable, i.e. the dots y-value is the respective closest multiple of k/n, where n is the group size and $k \in \{0, \dots n\}$. For PAM-orientation, both the uncertain predictions and the arrays for which no prediction was available from the dataset of Vink et al. are labeled as uncertain and thus contribute to the fractions.

In the CRISPRCasdb dataset, we found that many groups, e.g. the group shown in Figure 2, have high $|L_{\text{ratio}}|$ enabling *CRISPR-evOr* to make a clear decision for one orientation, when comparing both reconstructions.

However, for array groups where limited or erratic evolution results in lower likelihood ratios, decisions can be more difficult and not always obvious (Supplementary Fig. S3). Thus, we manually investigated the CRISPRCasdb data decisions with lower $|L_{\text{ratio}}|$ to determine a suitable conservative confidence threshold of $c = 5$, which indicates a difference of more than two orders of magnitude (e^5) between forward and reverse likelihoods. To further confirm the suitability of $c = 5$, we analyzed the difference in the number of deletion events between both reconstructions, as outlined in Methods.

However, we recommend that users conduct their own experiments and manual investigations to ensure that the threshold is reliable for their particular use case and parameter settings.

And we note in Methods "Confidence threshold c" (ll. 408 – 431):

For additional evidence that the threshold is suitable, we considered the difference in the number of deleted spacers between forward and reverse reconstructions for each group in the CRISPRCasdb dataset. In general, we find that the number of differences increases rapidly with increasing $|L_{\text{ratio}}|$ (Supplementary Fig. S4).

For $|L_{\text{ratio}}| = 0$, we typically do not have differences in the forward and backward reconstructions (see Supplementary Fig. S2). For $0 < |L_{\text{ratio}}| \leq 5$ the average difference in the number of spacer deletions between forward and backward is 5.5 (median 2). *CRISPR-evOr* still provides an estimate for the correct orientation in these cases. However, since the number of underlying deletion events is low, we believe that these results should be treated with caution. For $|L_{\text{ratio}}| > 5$, the average number of differences is 140.3 (median 42) with almost all groups exceeding at least 5. This is a substantial event count considering that arrays in the dataset have a median length of approximately 15 spacers. Combining both investigations leads us to believe that a conservative threshold of $c = 5$ allows a confident prediction of orientations.

Minor issues

1. Figure 3b, bottom stack, around 600 array count tick, the purple and blue bars are only PAM not confident. But the entire bottom stack should also be crispr-evor not confident?

A: *Thank you for noticing. The figure was indeed incorrectly labeled. We adjusted the y-axis labels to "confidence of methods" with "both confident" and "one or both not confident" assigned to the respective bars.*

2. Introduction, the comparison between "low confidence," "high confidence," and "available orientation predictions" is unclear. The distinction between low and high confidence makes sense and directly relates to method performance: CRISPR-evOr helps in low-confidence cases and tends to agree with other tools when high-confidence predictions are available. However, the "available orientation" category is conceptually distinct. If this refers to annotated orientations, which are used as ground truth or priors for the algorithm, this should be clearly stated and justified in the text. But based on the algorithm description, I don't think this is the case.

A: *You are correct, we never refer to experimentally annotated orientations. In the introduction "available orientation" means (high confidence) predictions by one of the other tools. We adjusted the introduction where "available" is used as follows (ll. 214 – 219):*

> Furthermore, on data where high confidence orientation predictions are already available, *CRISPR-evOr* helps to either reinforce the predictions, or resolve contradicting predictions of other tools, and thereby uncovers discrepancies among different orientation concepts dependent on the Cas type.

Additionally, "not available" is used in the context of the PAM dataset for parts of the dataset where the CRISPRCasdb dataset and Vink et al.'s dataset did not overlap and thus PAM-orientations are simply not available to us (e.g. in Supplementary Fig. S10, S14, S15). We state in Methods (ll. 278 – 280):

> Furthermore, only 38.9% (2309) of the arrays and 77.2% (400) of the groups in the CRISPRCasdb dataset have PAM-orientation predictions.

3. The discussion is overly long, please make it shorter

A: *We shortened the discussion wherever we believed it to be possible. Note that some parts of the discussion were extended through the moving of paragraphs from the results and as a result of the comments of Reviewer #2. Nonetheless the discussion contains more compact information now.*

Reviewer #2

This is a nice manuscript in which the authors develop and test a new tool, CRISPR-evOr, for detection of the direction of CRISPR arrays based on evolutionary history of spacer acquisition patterns. This can only works for arrays where several related CRISPR arrays are available, but in such cases the method can resolve low confidence or contradictory predictions from existing tools, which is very useful. My specific comments follow below.
Answer: Thank you very much for the kind and helpful comments and in-depth reading of the manuscript.

Issues

- "Moreover, CRISPR-evOr, 205 PAM-orientation, CRISPRDirection, and CRISPRstrand often disagree with each other for type II-C, which hints at differences between acquisition-orientation and leader-, repeat- and PAM-orientation for type II-C" – it is fairly well established that II-C systems often have a different spacer acquisition orientation than other systems which is at the leader distal rather than the leader proximal end. This should be

discussed, since it "derails" other tools also, but affects CRISPR-evOr the most (see for example the last paragraph of the Results. The authors only get to it in the discussion, where their interpretation is not clear enough for the reader to follow.

A: *We agree with your assessment. II-C has indeed been shown to have different acquisition order. Moreover, it has additional peculiarities like a different repeat mutation pattern and promoters within the repeats. This likely complicates orientation predictions based on leader and repeats. However, it should not influence the prediction of acquisition-orientation as long as the acquisition order is polarized and consistent within individual groups. Then regardless of the orientation with respect to transcription* CRISPR-evOr *is able to correctly infer the acquisition end. Although it can complicate the relation of acquisition-orientation with other orientation concepts which might result in the observed inconsistency with the repeat-based tools. Alternatively, the inconsistency of our tool could suggest that there might be diversity in acquisition-orientation for different II-C systems, i.e. subgroups of II-C types that have different acquisition behavior/orientation. We added a short section in the introduction as you suggested (ll. 159 – 166):*

> In particular, type II-C systems have been shown to be peculiar in multiple ways: they acquire spacers at the 3' end (opposite to the expected leader end) [22–24], carry promoters in the repeats [22, 24, 25] and show peculiar repeat mutation patterns [26] all of which could possibly interfere with accurate orientation inference.

and adjusted the discussion referencing to II-C to make our argumentation more clear (ll. 781 – 812):

> Type II-C has been shown to be particularly challenging to orient due to its unusual features in spacer acquisition, expression, leader, repeat structure [22, 24–26], and PAM diversity [11]. In particular, multiple studies reported that II-C arrays favor acquisition at the downstream 3'-end of the array [22–24].
>
> Moreover, Type II-C has not only leader promoters, but promoters in the repeats [22–25], which lowers the selective pressure to insert new spacers at the leader end and may cause deviations from patterns typically observed in leader and repeats of other CRISPR systems.
>
> However, as long as the (majority of) acquisitions are polarized and the acquisition end is consistent *within individual groups*, the predictions of *CRISPR-evOr* likely remain accurate and consistent. Indeed, manual inspection of the reconstructions of the groups of II-C arrays with confident predictions showed normal (polarized) evolutionary behavior and thus sensible acquisition-orientation predictions.
>
> Thus, the inconsistency of acquisition-orientation with both methods to predict repeat-orientation suggests that type II-C arrays might be composed of subgroups with different acquisition ends and thus inconsistency within (leader and) repeat structure. This could cause the complex discrepancies between acquisition-orientation and repeat-orientation.
>
> Multiple subtypes of II-C have been proposed in [4] based on the composition of their Cas loci and recent results in [47] point into the same direction, where we found that repeat diversity clusters II-C in multiple groups and could be related to co-occurrence of other Cas types. In any case, the inability of the tools to determine consistent orientations for type II-C warrants further investigation of the diversity and functionality of II-C systems.

- 372 " reconstruction process we identify and relabels unique" – fix

A: *Thank you. We resolved the typo.*

- 485 – fix ref (add number)

A: *We added the reference.*

Other corrections:

- We moved Figure 3a) to the supplementary. It is now Supplementary Fig. S3.
- We adjusted some formulations and corrected for typos.
